# Ultrasound texture‐based radiomics of placental and myometrial tissue for predicting postpartum blood loss in women with placenta previa and low‐lying placenta

**DOI:** 10.1111/aogs.70238

**Published:** 2026-05-04

**Authors:** Francesco Marasciulo, Elena Sofia Milandri, Ilaria Maffeo, Susanna Reghezza, Angelo Cagnacci, Ambrogio P. Londero, Federico Prefumo

**Affiliations:** ^1^ Unit of Obstetrics and Gynecology San Bortolo Hospital, ULSS n.8 “Berica” Vicenza Italy; ^2^ Unit of Obstetrics and Gynecology Santa Maria Delle Croci Hospital, AUSL Romagna Ravenna Italy; ^3^ Obstetrics and Gynecology Unit IRCCS Istituto Giannina Gaslini Genoa Italy; ^4^ Academic Unit of Obstetrics and Gynecology IRCCS Ospedale San Martino Genoa Italy; ^5^ Department of Neuroscience, Rehabilitation, Ophthalmology, Genetics, Maternal and Infant Health University of Genoa Genoa Italy

**Keywords:** placenta previa, postpartum hemorrhage, predictive modeling, radiomics, ultrasound

## Abstract

**Introduction:**

This study aimed to evaluate the role of radiomic analysis applied to ultrasound images in predicting postpartum blood loss at birth in women affected by low‐lying placenta or placenta previa.

**Material and Methods:**

In this retrospective, single‐center study, we analyzed singleton pregnancies with placenta previa or a low‐lying placenta, initially diagnosed at the second‐trimester ultrasound examination. Data were collected from ultrasound examinations conducted in the second and third trimesters, along with birth outcomes. Radiomic analysis was conducted on archival ultrasound images to extract quantitative features. Predictive models were constructed utilizing multivariable generalized linear modeling (Gamma regression with a log link), encompassing radiomics‐only, clinical/sonographic‐only, and an integrated model.

**Results:**

In the final analysis of 107 women, 51 exhibited postpartum blood loss exceeding 500 mL. A prior cesarean delivery was recognized as a notable clinical risk factor. Multiple radiomic features identified in second‐ and third‐trimester ultrasound scans correlated with a heightened risk of significant blood loss during birth. The integrated predictive model exhibited superior accuracy for blood loss exceeding 500 mL, achieving an AUC of 82.32% (95% CI: 74.18%–90.45%). This performance surpassed that of the clinical ultrasound model, which had an AUC of 71.27% (95% CI: 62.27%–80.27%), with a statistically significant difference (*p* = 0.001). Additionally, it demonstrated a nonsignificant improvement over the radiomics‐only model, which recorded an AUC of 77.17% (95% CI: 68.25%–86.09%).

**Conclusions:**

Radiomic analysis of ultrasound images enhances risk prediction for postpartum major blood loss in pregnancies affected by placenta previa and low‐lying placenta. Integrating radiomics with clinical and sonographic data improves predictive accuracy, offering a promising tool for personalized obstetric risk assessment and management.

## Graphical abstract

Radiomic analysis of ultrasound images combined with clinical data improves postpartum blood loss prediction in low‐lying placenta.

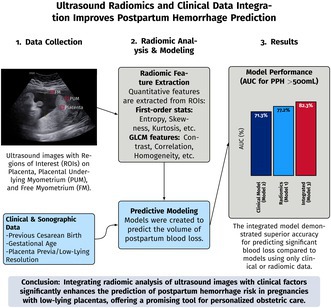

**Abbreviations**
ART: Assisted Reproductive Technology
AUC: Area Under the Curve
BMI: Body Mass Index
CB: Cesarean Birth
CI: Confidence Interval
FM: Free Myometrium
GLCM: Gray‐Level Co‐occurrence Matrix
ICC: Intraclass Correlation Coefficient
ICU: Intensive Care Unit
MRI: Magnetic Resonance Imaging
NICU: Neonatal Intensive Care Unit
PI: Pulsatility Index
PPH: Postpartum Hemorrhage
PUM: Placental Underlying Myometrium
ROC: Receiver Operating Characteristic
ROI: Region of Interest
SIEOG: Italian Society of Obstetric and Gynecological Ultrasound
VB: Vaginal Birth

**Key message**

Radiomic analysis of ultrasound images predicts postpartum blood loss risk in low‐lying placenta. Combining radiomic and clinical features improves blood loss prediction accuracy (AUC 82.32%). Ultrasound‐based radiomics offers accessible, personalized obstetric risk assessment.

## 1 INTRODUCTION

Implantation in the lower uterine segment characterizes the low‐lying placenta. Placentas are categorized according to their proximity to the internal cervical os: normally inserted (>20 mm), low‐lying (≤20 mm), or placenta previa (covering the internal os).^1^ The incidence of placenta previa at term is roughly 5.2 per 1000 pregnancies.^2^ Diagnosis generally occurs during standard fetal anomaly screening after 19 weeks of gestation.^3^ Spontaneous resolution is prevalent, occurring in approximately 90–95% of cases by the third trimester.^4^

Primary maternal risks linked to a low‐lying placenta encompass painless antepartum bleeding, which may result in hemorrhagic shock, transfusions, and the need for emergency interventions.^5^, ^6^ Neonatal complications predominantly arise from preterm birth, which increases the risks of perinatal morbidity and mortality.^7^ Massive hemorrhage, the most severe complication, presents a considerable challenge in the management of obstetric emergencies.^8^ The risk of spontaneous bleeding increases progressively throughout pregnancy, becoming particularly significant in the third trimester.^9^ Resolved low‐lying placentas identified in the second trimester continue to present an increased risk of postpartum bleeding.^10^, ^11^

Effective management of low‐lying and placenta previa requires dependable predictors of hemorrhage and tailored treatment approaches. There is a lack of clinical guidelines and standardized risk classifications for personalized management in cases of low‐lying placentas. Hemorrhage risk factors encompass prior cesarean birth, multiparity, advanced maternal age, history of placenta previa, previous uterine surgery, and smoking.^12^, ^13^, ^14^ Factors such as advanced maternal age and prior cesarean delivery increase the risk of placenta previa.^15^

**FIGURE 1:**
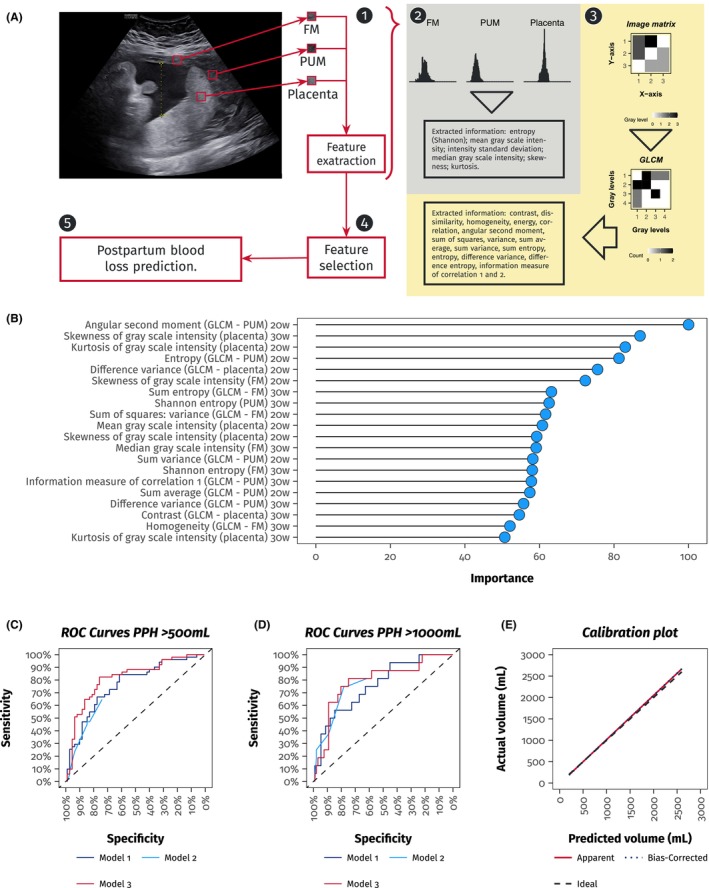
Panel (A) Workflow for the extraction of radiomic features and subsequent modeling. Regions of interest (ROIs) were manually delineated on the ultrasound image of the placenta and myometrium for each woman, encompassing the free myometrium (FM), the underlying myometrium (PUM), and the placenta (see step 1). Radiomic analysis was conducted on the gray‐scale pixel values within the defined ROIs. Initially, first‐order statistics, including mean and median intensity, standard deviation, skewness, kurtosis, and Shannon entropy, were computed from the intensity histogram to quantify the overall signal distribution (see step 2). Texture features were derived from gray‐level co‐occurrence matrices (GLCM), encompassing contrast, homogeneity, correlation, and additional second‐order descriptors that represent spatial relationships among adjacent pixels (see step 3). All extracted radiomic features were ranked based on their importance through a random forest‐based selection procedure (refer to Panel B) (see step 4), and the most informative features were retained. Ultimately, the selected radiomic features served as inputs for predictive modeling to estimate postpartum blood loss (in mL) and to evaluate the risk of postpartum hemorrhage (see step 5). Panel (B) This panel shows the variable importance plot from the random forest analysis. Radiomic features were ranked by their predictive importance for postpartum blood loss (in mL). Only the 15 most important features were retained for subsequent analysis. Panel (C) ROC curves of the multivariate models for predicting blood loss greater than 500 mL. The AUC of Model 1 was 77.17% (95% CI: 68.25%–86.09%), the AUC of Model 2 was 71.27% (95% CI: 62.27%–80.27%), and the AUC of Model 3 was 82.32% (95% CI: 74.18%–90.45%). The differences between Model 3 and Model 1, and between Model 3 and Model 2, were, respectively, *p* = 0.156 and *p* = 0.001. The difference between Model 2 and Model 1 was *p* = 0.305. Panel (D) ROC curves of the multivariate models for predicting blood loss greater than 1000 mL. The AUC of Model 1 was 76.79% (95% CI: 64.36%–89.21%), the AUC of Model 2 was 78.61% (95% CI: 65.78%–91.43%), and the AUC of Model 3 was 79.95% (95% CI: 67.34%–92.55%). The differences between Model 3 and Model 1, and between Model 3 and Model 2, were, respectively, *p* = 0.156 and *p* = 0.769. The difference between Model 2 and Model 1 was *p* = 0.809. Panel (E) Calibration Plot of the Multivariate Model (Model 3). Cross‐validation of Model 3, integrating radiomic and clinical features, demonstrates close alignment between predicted and actual probabilities of postpartum blood loss (in mL). The calibration plot indicates good predictive accuracy.

Radiomics is a developing quantitative imaging methodology that transforms standard medical images into high‐dimensional data through the extraction of features that characterize signal intensity, texture, shape, and complex patterns within a specified region of interest (ROI).^16^, ^17^ High‐throughput descriptors effectively capture subtle tissue heterogeneity that may be imperceptible to the human eye. These descriptors can be integrated with clinical variables in predictive models to enhance diagnosis, prognosis, and individualized risk prediction.^16^ A standardized radiomics workflow generally consists of image acquisition, segmentation, feature extraction, feature selection, and model construction (Figure 1A).^17^ In obstetric ultrasound, radiomics has been employed to analyze fetal lung texture for the non‐invasive prediction of neonatal respiratory morbidity and the assessment of fetal lung maturity. Parallel radiomics pipelines have been established using magnetic resonance imaging (MRI) in pregnancies affected by placenta previa and the placenta accreta spectrum (PAS) to assess the risk of peripartum hemorrhage.^18^, ^19^, ^20^, ^21^, ^22^ In this context, ultrasound texture‐based radiomics for placenta previa and low‐lying placenta may facilitate the assessment of postpartum hemorrhage (PPH) risk, utilizing the primary and most accessible imaging technique in obstetrics.^1^ This study evaluates radiomic patterns in second‐trimester ultrasound to predict postpartum bleeding in women with placenta previa and low‐lying placentas.

## 2 MATERIAL AND METHODS

### 2.1 Study design

This retrospective, single‐center study was conducted at a tertiary perinatal center and included women diagnosed with placenta previa or low‐lying placenta during second‐trimester ultrasound scans performed between January 1, 2020, and September 15, 2024. Enrolled women underwent an ultrasound scan between 19 and 23 + 6 weeks of gestation, with a diagnosis of placenta previa (placental implantation abutting or overlapping the internal cervical orifice) or low‐lying placenta (a distance of less than 20 mm from the placental edge to the internal cervical orifice).^23^ All women underwent standard ultrasound follow‐up in the third trimester to reevaluate placental position. Women who did not undergo subsequent ultrasound examinations following the second trimester or who delivered at different hospitals were excluded from the study.

### 2.2 Data collection

We collected each woman's anamnestic, clinical, and ultrasound data and extracted ultrasound images, labor, and birth outcomes. All ultrasound examinations were performed by experienced obstetricians using mid‐ to high‐range equipment, including the Voluson E6 (GE Healthcare, Zipf, Austria) and Samsung HERA W10 Elite (Samsung Medison, Seoul, South Korea) systems. Both transabdominal and transvaginal probes were used.

Ultrasound reports complied with national SIEOG guidelines for obstetric ultrasound, detailing the location of the placenta, its distance from the internal cervical os, and the presence of additional placental abnormalities, such as morphological anomalies, vasa previa, and signs of placental accreta.^24^ Each report included image documentation, facilitating the extraction of parameters including the distance from the placental edge to the internal cervical os, the presence or absence of the marginal sinus, the placental thickness 1 cm from the edge, or, in cases of placenta previa, the placental thickness above the internal os and the angle between the chorionic and basal plates.

Parameters were extracted for each ultrasound examination. Ultrasound reports and documentation were retrieved through the Astraia software system (NEXUS/ASTRAIA GmbH, Ismaning, Germany). Retrospective data regarding medical and obstetric history, pertinent clinical information, labor details, and neonatal outcomes were extracted from electronic medical records and reporting systems for all women included in the study.

### 2.3 Radiomic analysis

For each placental ultrasound image acquired during evaluations between 19 + 0 and 23 + 6 weeks and 28 + 0 and 32 + 6 weeks, a radiomic analysis was conducted. Images were extracted from the Astraia database in DICOM format. Manual delineation of ROIs was performed on the placenta, the placenta underlying myometrium (PUM), and placental‐free myometrium (FM) using ImageJ software (National Institutes of Health, Bethesda, USA). All ROIs had a standard size of 5 × 5 mm (25 mm^2^). Meta‐information and images were extracted from anonymized DICOM files before processing. The operators performing ROI placement were blinded to the clinical and outcome information of the cases. Quantitative radiomic parameters were extracted from each ROI, encompassing gray‐level histogram features, including Shannon entropy and characteristics derived from the gray‐level co‐occurrence matrix (GLCM). The GLCM was computed utilizing Python's Scikit‐image and Mahotas libraries (Python version 3.11.2), whereas gray‐level histogram analysis was conducted using R software (version 4.4.1; R Core Team 2024, R Foundation for Statistical Computing, Vienna, Austria). Figure 1A illustrates the overall workflow and parameters studied in the radiomic analysis. These parameters allowed for texture analysis of the images, capturing spatial relationships between pixels. This approach quantified variations in adjacent pixel intensities, describing the images' microstructural heterogeneity. All radiomic features were scaled using min‐max normalization. A correlation matrix was created to identify and exclude predictive variables with correlations greater than 0.9. Relevant radiomic features, for further analysis, were selected using a random forest plot to determine feature importance (model trained with 10‐fold cross‐validation and 3 repeats). Only the top 15 radiomic features were selected for further analysis. The reliability of radiomics was evaluated through intraclass correlation coefficients (ICC) to determine intra‐ and inter‐variability, following the interpretation guidelines established by Koo and Li^25^: ICC <0.50 indicates poor reliability, 0.50–0.75 indicates moderate reliability, 0.75–0.90 indicates good reliability, and >0.90 indicates excellent reliability.

### 2.4 Statistical analysis

Statistical analyses were performed using R software (version 4.4.1). Continuous variables were assessed for normality using the Kolmogorov–Smirnov test to determine whether they followed a parametric or non‐parametric distribution. In cases of non‐parametric distribution, continuous variables were summarized as median and interquartile range (IQR). Categorical variables were reported as absolute frequencies (numerator and denominator) and percentages. A priori power analysis for a general linear model determined that at least 54 participants are necessary to identify a medium effect size (Cohen's *f* ^2^ = 0.15) for a single predictor, achieving 80% power at an alpha level of 0.05.^26^ A total sample of 103 participants was required to power a full model incorporating up to seven independent variables.^26^ We utilized generalized linear models with a Gamma family and a log link to analyze the continuous, positive outcome of post‐partum blood loss, measured in milliliters, as the dependent variable. Initially, univariable Gamma regressions were performed. Potential predictors were subsequently evaluated through multivariable Gamma regression. Variables exhibiting a *p*‐value below 0.05 in the univariate analysis were included in the multivariate analysis, with final model variables determined via stepwise selection (AIC‐based). Given the exploratory, prediction‐oriented focus of this study, univariate *p*‐values were not adjusted for multiple comparisons, as they were intended solely for screening candidate variables rather than for confirmatory hypothesis testing. However, false discovery rate (FDR) correction was applied to the *p*‐values of the final multivariate models. The results of the Gamma regression are presented as univariate and multivariate mean ratios (MR) accompanied by 95% confidence intervals (95% CI). To compare predictive accuracy, we calculated the root mean squared error (RMSE) for each multivariate model. Model discrimination for hemorrhage thresholds (>500 mL and >1000 mL) was evaluated using receiver operating characteristic (ROC) curves and the area under the curve (AUC), with the Gamma regression‐predicted blood loss serving as the continuous predictor. The DeLong's test was employed to evaluate the differences between the two AUCs. To address optimism, we conducted resampling validation of the fitted model's AUCs through 1000 random bootstrap resamples. Each iteration involved refitting the same Gamma‐log link model on a bootstrap draw, calculating AUC for both the bootstrap sample (optimistic performance) and the original sample, and documenting the difference as the optimism estimate for that iteration. The mean optimism from all resamples was deducted from the apparent AUC to produce an optimism‐corrected AUC. A 95% CI for the corrected AUC was established using the percentile method based on the distribution of AUCs obtained from the bootstrap procedure. The calibration plot includes regression from the original data alongside bias‐corrected predicted values obtained through the bootstrap procedure. A significance level of *p* < 0.05 was applied for all analyses.

## 3 RESULTS

During the study period, 166 singleton pregnancies with placenta previa or low‐lying placenta diagnoses were evaluated. Twenty women were lost (12.1%) to ultrasound follow‐up, and 29 women delivered in other hospitals (17.5%), resulting in a final cohort in our analysis of 107 women. In singleton pregnancies with accessible follow‐up data, placenta previa or low‐lying placenta resolved in 84% of instances, with a median resolution gestational age of 30.14 weeks (IQR, 30–30.57).

The median blood loss following birth was 400 mL (IQR, 300.00/700.00 mL). In total, 51 women experienced blood loss of more than 500 mL, and 16 had blood loss of more than 1000 mL. Among pregnancy‐ and delivery‐related factors, previous cesarean birth emerged as a strong risk marker (MR 1.96; 95% CI 1.35–2.86; *p* < 0.05), whereas resolved placenta previa/low‐lying (MR 0.54; 95% CI 0.38–0.77; *p* < 0.05) was protective (Table 1). Instrumental vaginal delivery (vacuum) and both planned (MR 2.27; 95% CI 1.63–3.16; *p* < 0.05) and emergency cesarean birth (MR 1.93; 95% CI 1.33–2.82; *p* < 0.05) were each linked to significantly greater blood loss compared with spontaneous vaginal birth. Increased bleeding was associated with maternal admission to the intensive care unit and lower postpartum hemoglobin (Table 1). With respect to neonatal and gestational parameters, each additional week of gestational age reduced mean maternal blood loss by 10% (MR 0.90 per week; 95% CI 0.83–0.98; *p* < 0.05). No significant associations were observed for neonatal sex, birth weight, Apgar scores, or NICU admission. Table 2 presents the ultrasound characteristics at enrollment. On ultrasound at enrollment, placenta previa (any overlap of the placental edge with the internal os) was present in 35.5% of women and was associated with a 37% increase in mean blood loss at delivery compared with low‐lying placenta (MR 1.37; 95% CI 1.01–1.86; *p* < 0.05) and was significantly and inversely correlated with placenta previa/low‐lying resolution (Table 2). No other sonographic feature was significantly associated with blood loss (Table 2).

**TABLE 1:** Characteristics of women, pregnancies, and neonates are reported as mean (± standard deviation), median, IQR, or as percentages and absolute values.

Variables	Values	Mean Ratio (95% CI)	*p*
Woman characteristics			
Age (years)	34.40 (±4.78)	1.03 (1.00/1.06)	0.097
BMI (kg/m^2^)	22.84 (20.13/25.54)	1.01 (0.98/1.05)	0.460
Ethnic group			
Caucasic	90.65% (97/107)	Reference	–
Hispanic	5.61% (6/107)	0.85 (0.44/1.64)	0.622
Other	3.74% (4/107)	1.70 (0.77/3.78)	0.195
Diabetes mellitus			
No	84.11% (90/107)		
DM1	1.87% (2/107)	0.94 (0.30/2.92)	0.917
dGDM	12.15% (13/107)	1.35 (0.84/2.15)	0.216
iGDM	1.87% (2/107)	1.79 (0.58/5.54)	0.316
Tobacco smoke	6.60% (7/106)	1.22 (0.67/2.25)	0.516
Nulliparous	50.47% (54/107)	0.92 (0.69/1.24)	0.605
Nulligravid	38.32% (41/107)	0.88 (0.65/1.19)	0.413
Previous PPH	0.93% (1/107)	2.35 (0.49/11.29)	0.289
Mode of conception			
Spontaneous	84.11% (90/107)	Reference	–
Ovulation induction	0.93% (1/107)	1.48 (0.30/7.34)	0.634
ART	14.95% (16/107)	1.21 (0.79/1.87)	0.384
Previous surgery			
Previous CB	14.95% (16/107)	1.96 (1.35/2.86)	<0.05
Previous dilation and curettage	22.43% (24/107)	0.89 (0.62/1.28)	0.522
Previous hysteroscopy	7.48% (8/107)	0.96 (0.54/1.70)	0.876
Previous instrumental removal of the placenta	1.87% (2/107)	1.39 (0.46/4.25)	0.562
Previous myomectomy	1.87% (2/107)	0.89 (0.29/2.71)	0.838
Pregnancy characteristics			
Admission during pregnancy	8.41% (9/107)	1.70 (0.97/2.97)	0.065
Admission for bleeding	5.61% (6/107)	1.60 (0.82/3.11)	0.171
Hospitalization without birth	6.54% (7/107)	1.65 (0.88/3.07)	0.119
Placenta previa/low‐lying resolution	79.44% (85/107)	0.54 (0.38/0.77)	<0.05
Labor induction	47.44% (37/78)	1.08 (0.77/1.50)	0.665
Postpartum blood loss (mL)^a^	400 (300/700)	–	–
Blood loss >500 mL^a^	47.66% (51/107)	–	–
Blood loss >1000 mL^a^	14.95% (16/107)	–	–
Hemoglobin before birth (g/dL)	11.16 (±1.09)	1.06 (0.84/1.34)	0.640
Hemoglobin after birth (g/dL)	10.67 (±1.34)	0.70 (0.64/0.77)	<0.05
Maternal admission in ICU	4.72% (5/106)	2.72 (1.38/5.33)	<0.05
Mode of delivery			
VB	50.47% (54/107)	Reference	–
Vacuum	8.41% (9/107)	2.13 (1.30/3.52)	<0.05
CB (planned)	24.30% (26/107)	2.27 (1.63/3.16)	<0.05
CB (emergency)	16.82% (18/107)	1.93 (1.33/2.82)	<0.05
Neonatal characteristics			
Neonatal sex			
Female	45.65% (42/92)	Reference	–
Male	54.35% (50/92)	1.16 (0.82/1.63)	0.412
Gestational age at birth (weeks)	39.00 (38.00/40.07)	0.90 (0.83/0.98)	<0.05
Gestational age at birth (<37 weeks)	7.48% (8/107)	1.69 (0.94/3.02)	0.082
Birth weight (g)	3173.12 (±497.99)	1.00 (1.00/1.00)	0.332
Birth weight (centiles)	47.28 (±25.99)	1.00 (1.00/1.01)	0.551
Apgar score (1st min)	9.00 (8.25/9.00)	0.88 (0.63/1.25)	0.485
Apgar score (5th min)	10.00 (9.00/10.00)	0.88 (0.58/1.33)	0.547
NICU admission	7.61% (7/92)	1.53 (0.83/2.84)	0.177

*Note*: The final two columns present the associations between woman, pregnancy, and neonatal characteristics and blood loss at birth (in mL), estimated using generalized linear models with a Gamma distribution and log link. Effect estimates are reported as MRs with 95% confidence intervals (95% CI). For a continuous predictor, an MR of 1.03 indicates a 3% increase in expected mean blood loss per 1‐unit increase (e.g., each additional year of maternal age). For a binary predictor, an MR of 1.96 indicates 96% higher expected mean blood loss when the characteristic is present versus absent (e.g., previous cesarean birth: Yes vs. no).

Abbreviations: ART, assisted reproductive technology; BMI, body mass index; CB, cesarean birth; DM1, type 1 diabetes mellitus; dGDM, diet‐controlled gestational diabetes mellitus; iGDM, insulin‐treated gestational diabetes mellitus; ICU, intensive care unit; IQR, interquartile range; MR, mean ratio; NICU, neonatal intensive care unit; PPH, postpartum hemorrhage; VB, vaginal birth.

^a^ Blood loss was the outcome variable of the model; therefore, MR values are not applicable for the rows describing blood loss distribution.

**TABLE 2:** Ultrasound assessment during enrollment and quantification of blood loss at delivery.

Variables	Values	Mean Ratio (95% CI)	*p*
Placenta location at enrollment			
Low lying at enrollment	64.49% (69/107)	Reference	–
Previa at enrollment	35.51% (38/107)	1.37 (1.01/1.86)	<0.05
Visible marginal sinus of the placenta	20.24% (17/84)	1.40 (0.95/2.08)	0.096
Marginal sinus over the internal os	57.14% (8/14)	1.48 (0.67/3.24)	0.350
Vasa previa	0.94% (1/106)	1.79 (0.37/8.63)	0.471
Placenta over the internal os	31.78% (34/107)	1.35 (0.99/1.84)	0.062
Anterior placenta	39.25% (42/107)	0.85 (0.63/1.17)	0.324
Placental anomalies			
No abnormalities	97.20% (104/107)	Reference	–
Bilobed or succenturiate placenta	2.80% (3/107)	2.21 (0.89/5.52)	0.091
Distance from the placental margin and internal os (mm)	10.73 (±4.79)	1.00 (0.96/1.03)	0.841
Distance from the marginal sinus and internal os (mm)	5.83 (±5.95)	1.01 (0.91/1.12)	0.910
Placenta thickness (mm)	13.00 (9.00/18.00)	1.01 (0.99/1.02)	0.339
Basal plate − chorionic plate angle (degree)	68.45 (±23.67)	1.00 (0.99/1.01)	0.813
Cervical length (mm)	40.00 (36.00/44.25)	0.99 (0.97/1.02)	0.561

*Note*: The characteristics of the ultrasound examination at enrollment are reported as mean (± standard deviation), median (IQR), or as percentages and absolute values. The last two columns present the relationships between ultrasound examination characteristics at enrollment and blood loss at birth (in mL), analyzed using generalized linear models with a Gamma distribution and log link. Effect estimates are reported as MRs with 95% CI. For a continuous predictor, an MR of 1.03 indicates a 3% increase in expected mean blood loss per 1‐unit increase (e.g., each additional year of maternal age). For a binary predictor, an MR of 1.96 indicates 96% higher expected mean blood loss when the characteristic is present versus absent (e.g., previous cesarean birth: Yes vs. no).

Abbreviations: 95% CI, 95% confidence interval; IQR, interquartile range; MR, mean ratio.

The intra‐operator ICC agreement for the radiomic analysis was 0.94 (95% CI 0.93–0.95), and the inter‐operator ICC agreement was 0.90 (95% CI 0.88–0.92). A preliminary analysis of radiomic feature importance was performed using a random forest algorithm (Figure 1B). Of the top‐15 radiomic features evaluated, five showed significant associations with blood loss at delivery. Greater skewness of placental gray‐scale intensity at 30 weeks was linked to higher mean blood loss (MR 2.42; 95% CI 1.17–5.00; *p* < 0.05), as was a higher information measure of correlation‐1 in the placental underlying myometrium at 30 weeks (MR 2.44; 95% CI 1.06–5.62; *p* < 0.05) (Table 3). In contrast, increased Shannon entropy of the placental underlying myometrium at 30 weeks was protective (MR 0.37; 95% CI 0.14–0.98; *p* < 0.05), as were higher mean gray‐scale intensity in the placenta at 20 weeks (MR 0.43; 95% CI 0.21–0.86; *p* < 0.05) and higher median gray‐scale intensity in the free myometrium at 30 weeks (MR 0.34; 95% CI 0.17–0.70; *p* < 0.05). Among these significant features, one is from FM ROIs, two are from placenta ROIs, and two are from PUM ROIs. One feature was derived from 20‐week ultrasound scans, and four from 30‐week scans.

**TABLE 3:** Relationships between the top 15 radiomic parameters and blood loss at birth (in mL), analyzed using generalized linear models with a Gamma distribution and log link.

Variables	Mean Ratio (95% CI)	*p*
Angular second moment (GLCM − PUM) 20w	2.58 (0.83/8.03)	0.104
Skewness of gray‐scale intensity (placenta) 30w	2.42 (1.17/5.00)	<0.05
Kurtosis of gray‐scale intensity (placenta) 20w	1.06 (0.41/2.77)	0.905
Entropy (GLCM − PUM) 20w	0.73 (0.29/1.85)	0.507
Difference variance (GLCM − placenta) 20w	1.98 (0.92/4.28)	0.085
Skewness of gray‐scale intensity (FM) 20w	0.97 (0.43/2.19)	0.948
Sum entropy (GLCM − FM) 30w	1.03 (0.42/2.56)	0.941
Shannon entropy (PUM) 30w	0.37 (0.14/0.98)	<0.05
Sum of squares: variance (GLCM − FM) 20w	1.51 (0.55/4.12)	0.423
Mean gray‐scale intensity (placenta) 20w	0.43 (0.21/0.86)	<0.05
Skewness of gray‐scale intensity (placenta) 20w	1.82 (0.64/5.14)	0.261
Median gray scale intensity (FM) 30w	0.34 (0.17/0.70)	<0.05
Sum variance (GLCM − PUM) 20w	0.54 (0.16/1.79)	0.317
Shannon entropy (FM) 30w	0.58 (0.22/1.55)	0.281
Information measure of correlation 1 (GLCM − PUM) 30w	2.44 (1.06/5.62)	<0.05

*Note*: Effect estimates are reported as MR with 95% CI. For a continuous predictor, an MR of 1.03 indicates a 3% increase in expected mean blood loss per 1‐unit increase (e.g., each additional year of maternal age). For a binary predictor, an MR of 1.96 indicates 96% higher expected mean blood loss when the characteristic is present vesrus absent (e.g., previous cesarean birth: Yes vs. no).

Abbreviations: 95% CI, 95% confidence interval; FM, free myometrium; GLCM, gray‐level co‐occurrence matrix; MR, mean ratios; PUM, placental underlying myometrium; w, weeks of gestation.

Table 4 presents the characteristics of the three most predictive multivariate models. The independent variable was defined as blood loss (in mL). The initial model (Model 1) incorporated solely radiomic variables, achieving an AUC of 77.17% (95% CI: 68.25%–86.09%) for blood loss >500 mL. The second model (Model 2) integrated anamnestic and ultrasound variables, achieving an AUC of 71.27% (95% CI: 62.27%–80.27%) for blood loss >500 mL. The third model (Model 3) integrated anamnestic, ultrasound, and radiomic variables, achieving an AUC of 82.32% (95% CI: 74.18%–90.45%) for blood loss >500 mL. After FDR correction, the key predictors in each model retained consistent effect estimates (Table 4). Figure 1C illustrates the ROC curves for the three models, with the AUC differences between Model 3 and Model 1 or Model 2 being *p* = 0.156 and *p* = 0.001, respectively. The same pattern of AUC differences was found for blood loss >1000 mL, but without significant differences between the models (Figure 1D). The Model 3, 2, and 1 RMSEs were respectively 364.71, 377.03, and 392.64. Cross‐validation was performed for Model 3 (Figure 1E), showing an optimism‐corrected AUC of 80.37% (95% CI: 78.05%–82.74%) for blood loss >500 mL and of 76.50% (95% CI: 75.06%–80.36%) for blood loss >1000 mL. The calibration plot was also evaluated in Figure 1D (Model 3), showing the apparent calibration curve (slope 1.03, intercept −17.50) deviates modestly from the ideal line (slope 1.00, intercept 0), and the bias correction remains close to the ideal line (slope 1.04, intercept −21.59).

**TABLE 4:** Multivariate models for blood loss prediction at birth (in mL).

Variables	Mean Ratio (95% CI)	*p*	Mean Ratio (95% CI)(*)	*p*(*)	*p*(**)
Blood loss (radiomics) (Model 1)					
Skewness of gray scale intensity (placenta) 30w	2.42 (1.17/5.00)	<0.05	2.04 (1.03/4.07)	<0.05	0.059
Shannon entropy (PUM) 30w	0.37 (0.14/0.98)	<0.05	0.46 (0.19/1.12)	0.089	0.089
Mean gray‐scale intensity (placenta) 20w	0.43 (0.21/0.86)	<0.05	0.47 (0.24/0.91)	<0.05	0.055
Median gray‐scale intensity (FM) 30w	0.34 (0.17/0.70)	<0.05	0.35 (0.18/0.67)	<0.05	<0.05
Information measure of correlation 1 (GLCM − PUM) 30w	2.44 (1.06/5.62)	<0.05			
Blood loss (other variables) (Model 2)					
Age (years)	1.03 (1.00/1.06)	0.097			
BMI (kg/m^2^)	1.01 (0.98/1.05)	0.46			
ART	1.21 (0.79/1.85)	0.393			
Previous PPH	2.35 (0.49/11.29)	0.289			
Nulliparous	0.92 (0.69/1.24)	0.605			
Placenta thickness (mm)	1.01 (0.99/1.02)	0.339			
Mean uterine arteries PI	1.15 (0.81/1.65)	0.442			
Labor induction	1.08 (0.77/1.50)	0.665			
Gestational age at birth (weeks)	0.90 (0.83/0.98)	<0.05			
Birth weight (centiles)	1.00 (1.00/1.01)	0.551			
Anterior placenta	0.85 (0.63/1.17)	0.324			
Previous CB	1.96 (1.35/2.86)	<0.05	1.57 (1.05/2.35)	<0.05	0.079
Cesarean birth	1.83 (1.39/2.43)	<0.05	1.36 (0.97/1.90)	0.079	0.079
Placenta previa/low‐lying resolution	0.54 (0.38/0.77)	<0.05	0.70 (0.48/1.03)	0.073	0.079
Blood loss (all variables) (Model 3)					
Skewness of gray‐scale intensity (placenta) 30w	2.42 (1.17/5.00)	<0.05	1.88 (0.98/3.60)	0.06	0.099
Shannon entropy (PUM) 30w	0.37 (0.14/0.98)	<0.05			
Mean gray‐scale intensity (placenta) 20w	0.43 (0.21/0.86)	<0.05	0.67 (0.35/1.30)	0.241	0.241
Median gray‐scale intensity (FM) 30w	0.34 (0.17/0.70)	<0.05	0.41 (0.21/0.78)	<0.05	<0.05
Information measure of correlation 1 (GLCM − PUM) 30w	2.44 (1.06/5.62)	<0.05			
Age (years)	1.03 (1.00/1.06)	0.097			
BMI (kg/m^2^)	1.01 (0.98/1.05)	0.46			
ART	1.21 (0.79/1.85)	0.393			
Previous PPH	2.35 (0.49/11.29)	0.289			
Nulliparous	0.92 (0.69/1.24)	0.605			
Placenta thickness (mm)	1.01 (0.99/1.02)	0.339			
Mean uterine arteries PI	1.15 (0.81/1.65)	0.442			
Labor induction	1.08 (0.77/1.50)	0.665			
Gestational age at birth (weeks)	0.90 (0.83/0.98)	<0.05			
Birth weight (centiles)	1.00 (1.00/1.01)	0.551			
Anterior placenta	0.85 (0.63/1.17)	0.324			
Previous CB	1.96 (1.35/2.86)	<0.05	1.43 (0.96/2.12)	0.084	0.105
Cesarean birth	1.83 (1.39/2.43)	<0.05	1.50 (1.12/2.01)	<0.05	<0.05
Placenta previa/low‐lying resolution	0.54 (0.38/0.77)	<0.05			

*Note*: Associations between radiomic and clinical predictors and blood loss at birth were estimated through generalized linear models utilizing the Gamma family with a log link function. Data are presented as univariate and multivariate MRs with 95% CI. For a continuous predictor, an MR of 1.03 indicates a 3% increase in expected mean blood loss per 1‐unit increase (e.g., each additional year of maternal age). For a binary predictor, an MR of 1.96 indicates 96% higher expected mean blood loss when the characteristic is present versus absent (e.g., previous cesarean birth: Yes vs. no). Columns marked with (*) report multivariate effect estimates and corresponding *p*‐values obtained after AIC‐based stepwise variable selection. Columns marked with (**) report *p*‐values adjusted for multiple comparisons using the FDR correction.

Abbreviations: ART, assisted reproductive technology; BMI, body mass index; CB, cesarean birth; 95% CI, 95% confidence interval; FM, free myometrium; GLCM, gray‐level co‐occurrence matrix; MR, mean ratio; PPH, postpartum hemorrhage; PUM, placental underlying myometrium; w, weeks of gestation.

## 4 DISCUSSION

In our cohort, three quantitative ultrasound textural features were independently associated with postpartum blood loss: the skewness of gray scale intensity of the placenta at 30 weeks, the median gray scale intensity of the free myometrium at 30 weeks, and the mean gray scale intensity of the placenta at 20 weeks. A predictive model that integrates radiomic markers with clinical and sonographic covariates (Model 3) demonstrated superior discrimination for hemorrhage exceeding 500 mL.

Our analysis identified previous cesarean birth as a statistically significant factor associated with increased postpartum blood loss, aligning with prior findings.^12^, ^14^ Other clinical and ultrasound parameters exhibited negligible, nonsignificant differences. Recent literature links increased placental thickness to PPH^27^; our study similarly observed a slight increase in thickness in cases with greater blood loss, although this finding was not statistically significant.

Labor and birth outcomes aligned with the current body of evidence.^5^, ^7^, ^28^ Women with higher blood loss were more commonly delivered via cesarean section, had earlier gestational ages at birth, demonstrated secondary anemia, and required intensive care admission more frequently. Neonatal outcomes were similar, with the exception of a greater incidence of preterm birth in the high blood loss women.

Akazawa and Hashimoto indicate that clinical risk factors, including previous cesarean sections and maternal age, underscore general hemorrhage risks; however, they do not provide specific placental details.^21^ Standard ultrasound provides qualitative evaluations of the placenta, encompassing its position and conditions such as placenta previa, low‐lying placenta, and PAS disorders. Radiomics offers a quantitative method for ultrasound assessment, improving the diagnosis and management of placenta previa.

Radiomic analysis, a recent advancement in medical imaging, has primarily been utilized in MRI within obstetrics. While standardized acquisition protocols enhance feature extraction, they also restrict routine clinical applicability due to cost and availability limitations.^21^ Since MRI is not routinely required for the management of placenta previa, there is a pressing need to validate ultrasound‐based radiomic tools that can be embedded in standard care pathways. These tools can improve diagnostic and therapeutic planning by combining quantitative image analysis with traditional sonographic and clinical data.

Recent studies utilizing MRI‐based radiomics have concentrated on the antenatal prediction of PPH, especially in women diagnosed with placenta previa and/or PAS. Wu et al. created an initial clinicoradiomic nomogram utilizing placental T2‐weighted imaging (T2WI), which successfully differentiated between PPH and demonstrated high sensitivity for severe PPH in both internal and external cohorts.^18^ Further studies involving cohorts with placenta previa and suspected PAS have validated that radiomics signatures, when integrated with clinical variables, can effectively predict significant blood loss, demonstrating high AUC values and outperforming models based solely on clinical factors.^19^, ^20^, ^21^, ^29^ Recent advancements have introduced fusion models that combine radiomics with 2D/3D deep‐learning features. Late‐fusion architectures have demonstrated optimal performance and robustness across both subgroups, with and without placenta previa/PAS.^22^ Imaging‐based approaches enhance existing PPH risk‐prediction methods, which have historically relied on clinical and intrapartum variables and have excluded placental imaging.^30^, ^31^ MRI radiomics offers comprehensive placental texture data on standardized T2WI; however, its application is predominantly limited to specific high‐risk cases.

Elevated postpartum blood loss is significantly linked to adverse maternal outcomes, such as increased rates of intensive care unit admission. Ultrasound texture‐based radiomics serves as a more accessible, first‐line, real‐time method for risk stratification of postpartum hemorrhage in women with placenta previa or low‐lying placenta, especially in environments where MRI is not commonly available. This study builds on MRI radiomics evidence by proposing an integrated ultrasound‐based approach that combines radiomic features with clinical and sonographic variables (Model 3). The findings demonstrate optimal predictive performance and highlight the value of multimodal information for individualized risk assessment.

This study's strengths encompass, for the first time, the application of radiomic analysis utilizing accessible ultrasound technology, the execution of longitudinal radiomic assessments at 20 and 30 weeks of gestation, and the creation of multivariate predictive models. A further strength is the robust intra‐ and inter‐observer reproducibility of the radiomic workflow (ICC, 0.94 and 0.90, respectively). The assessment of the reproducibility of radiomic features derived from archived ultrasound images primarily considers how consistently ROIs are placed and how features are extracted from different stored images. However, evaluating the overall reproducibility (test–retest) that includes variability from the image acquisition stage, such as repeated scans, probe settings, and differences in operator technique, was not possible. To effectively measure the full reproducibility from image acquisition to radiomics analysis, standardized acquisition protocols and repeated scans in prospective studies are essential. This study employed a radiomic approach to analyze placental tissue, subplacental myometrium, and myometrium distant from the placenta, thereby improving data precision and offering potential insights into placental attachment abnormalities. This approach enables the development of tailored clinical interventions suitable for diverse contexts.

This study presents several limitations. The retrospective design and small sample size limit the generalizability of the findings. The proposed models were developed through a retrospective single‐center cohort and subjected to internal bootstrap validation. Nonetheless, external validation using an independent dataset remains necessary to establish generalizability across various centers, devices, and acquisition settings. Future multicenter studies should prospectively evaluate the model and assess its performance stability under both standardized and real‐world acquisition conditions. Radiomic analysis used manual segmentation, which could have introduced observational bias compared with automated methods.^16^, ^17^ However, the intra‐ and inter‐observer reproducibility demonstrated strong results, with observers blinded to clinical outcomes. Variability in women's characteristics, ultrasound devices, and image acquisition presents further limitations. High reproducibility alleviated concerns despite the use of archived images from various devices and operators. Future studies employing advanced ultrasound technology and standardized protocols are essential to validate these findings and improve the reliability of radiomic predictions for postpartum blood loss.

The integration of ultrasound‐based radiomic analysis in prenatal care has the potential to enhance the early identification of women at elevated risk for postpartum hemorrhage, facilitating tailored clinical management concerning surveillance, timing, and birth location. Future multicenter studies are crucial for validating these models in larger and more diverse populations. Ultrasound radiomics can enhance the understanding of myometrial structure and dysfunction, thereby elucidating the mechanisms underlying pregnancy complications. This study is the first to demonstrate the feasibility and utility of ultrasound‐based radiomic assessment of placental and myometrial tissues.

## 5 CONCLUSION

In our cohort, 84.3% of women with abnormal placental implantation at 20 weeks demonstrated resolution. The radiomic pattern features of the placenta, placental‐underlying myometrium, as well as free myometrium features, were independently associated with the risk of bleeding postpartum. The multivariate model for predicting significant blood loss (>500 mL) demonstrated superior accuracy compared to the clinical model, achieving an AUC of 82.32%.

## AUTHOR CONTRIBUTIONS

Substantial contributions to conception and design or acquisition of data or to analysis and interpretation of data (FM, ESM, IM, SR, AC, APL, FP). Drafting the article or revising it critically for important intellectual content (FM, ESM, IM, SR, AC, APL, FP). All authors have read and approved the final manuscript.

## FUNDING INFORMATION

This study has had no financial support.

## CONFLICT OF INTEREST STATEMENT

The authors declare that they have no potential conflicts of interest relevant to this article.

## ETHICS STATEMENT

The local ethical committee approved the data analysis and publication (CET Liguria on November 23, 2023; registry number 276/2023‐DB id 13212). All women signed informed consent forms to allow their clinical data to be used anonymously in scientific publications. This investigation was carried out following the instructions of national health authorities and good clinical practice procedures.

## Data Availability

The data that support the findings of this study are available. However, restrictions apply to the availability of these data, which were used under license for the current study and are not publicly available. Data are, however, available from the authors upon reasonable request and with permission of the Internal Review Board.
